# Editorial: Factors and health outcomes of job burnout

**DOI:** 10.3389/fpubh.2022.1023462

**Published:** 2022-11-08

**Authors:** Angela Stufano, John Koku Awoonor-Williams, Luigi Vimercati

**Affiliations:** ^1^Interdisciplinary Department of Medicine, University of Bari Aldo Moro, Bari, Italy; ^2^Formerly Ghana Health Service Policy Planning, Monitoring, and Evaluation Division, Accra, Ghana; ^3^Health System Policy Analyst and Independent Public Health Consultant, Accra, Ghana

**Keywords:** burnout, wellbeing, mental health, work related stress, emotional intelligence, healthcare workers, firefighters, nurses

Burnout is a psychological syndrome caused by prolonged exposure to chronic interpersonal stressors in a job. The 11th revision of the International Classification of Diseases, published in 2019 by the World Health Organization, includes burnout syndrome as an occupational phenomenon in the chapter on factors affecting health status or contact with health services (1, 2). According to Maslach and Jackson, burnout syndrome is defined by four key factors: overwhelming exhaustion, feelings of cynicism, detachment from work, and a sense of ineffectiveness and lack of achievement (1, 2). The prevalence of burnout syndrome in the helping professions (such as teachers or clinical staff) seems to be higher than in other occupations. However, this phenomenon has also become relevant in other occupational sectors that are characterized by high levels of work-related stress.

Job burnout is a subject that has triggered widespread interest among the general public and the media, and has been the subject of research and policy responses across Europe. While some work-related psycho-social risks such as heavy workload, long working hours, and overtime seem to trigger burnout, the influence of other factors such as autonomy, the degree of influence of management, and the role of rewards is more ambiguous. Without detection and proper treatment, burnout symptoms can be chronic. Preventive actions include checklists and tools to aid early detection, training programs for high-risk jobs, awareness-raising actions, and good-practice guidelines (3). In addition, the significant increase in the prevalence of this syndrome in different occupational settings has been associated with the recent COVID-19 pandemic, particularly for healthcare workers, who are at a high risk of exposure to infection (4–7) and several psycho-social and work-related risk factors (8). Based on this, it is of paramount importance to identify workers who are prone to psychological disorders, and to design supportive interventions that promote wellbeing and coping in occupational settings which could be integrated into overall health, social, and economic care management in the current pandemic (9).

Healthcare is perceived as one of the most stressful work environments, as it requires intense personal interactions with patients and colleagues. Burnout is therefore a serious concern among healthcare workers (HCWs), and has received increased attention in recent years. In particular, the COVID-19 pandemic appears to have exacerbated mental health problems in healthcare workers; they were found to be more prone to depression, anxiety, stress, and insomnia, and in many cases may experience long-term effects following SARS-CoV-2 infection (10). The increased focus on burnout in HCWs also stems from negative consequences on patient safety, non-uniformity of care, healthcare system costs, and workflow, as well as the lack of safety and health monitoring for the workers themselves. Furthermore, a significant relationship between the risk of medical errors and burnout has been observed (Alijabri et al.).

The extremely high number of cases and deaths during the COVID-19 pandemic, and the need to implement new containment strategies to limit the spread of the infection, have increased awareness of the already difficult working conditions faced by healthcare workers (11). Therefore, the identification of new strategies to deal with burnout syndrome, by identifying possible solutions, is crucial (Leo et al.). Several factors seem to be associated with the incidence of burnout in HCWs. The importance of emotional intelligence as a protective factor against burnout suggests that lifestyle modifications contribute to an increase in emotional intelligence (Sharaf et al.). Therefore, emotional intelligence, as a measurable positive psychological resource and a key non-technical skill in mitigating burnout levels in HCWs, should receive increased attention (Cao et al.). Furthermore, it has been hypothesized that the management of physicians' workload related to paperwork during outpatient encounters could be of great importance in decreasing the risk of burnout, promoting physicians' physical and psychological wellbeing, and improving the quality of physician-patient interactions (Li et al.). Another factor related to a higher incidence of burnout, particularly during the pandemic, was the presentism rate of medical personnel, which is much higher than for other jobs; while saving the lives of others, medical personnel were more likely to neglect their own health (Jia et al.). Finally, the COVID-19 pandemic has forced millions of people worldwide to adopt remote working environments using a variety of online platforms, increasing the prevalence of technostress as a possible source of burnout, and highlighting negative emotional responses to technology, including those in medical students and residents (Kasemy et al.).

Among HCWs, a job group that is particularly exposed to burnout is nurses. Research has revealed how various working conditions such as an exhaustive job, being moved between different patient care units within an organization, high job demands, and ineffective relationships with co-workers, supervisors, and/or physicians may produce higher levels of burnout (Feleke et al.). Moreover, other conditions such as workplace bullying among nurses and its relationship with organizational culture, the avoidance of a conflictive management style, low job satisfaction, and increased nurse-patient ratios have also been identified as burnout facilitators (12). Job satisfaction can be influenced by both psycho-social and organizational work factors. Consequently, improving nurses' specific job characteristics such as autonomy or nurse–physician collaboration could help to maintain an adequate degree of engagement in their job (Sanso et al.). Moreover, the spread of the COVID-19 pandemic and the increased number of cases and admissions have greatly influenced the wellbeing of hospital nurses, who may experience burnout due to physical and psychological work demands that exceed their ability to handle them (Kangarlou et al.). In particular, certain factors might have influenced the prevalence of burnout in these workers during the COVID-19 pandemic, for example, sickness presentism, which is considered a symbol of traditional dedication and diligence particularly in Eastern cultures. This can negatively affect nurses' physical and mental health, productivity, and work quality, and can increase the incidences of fatigue and job burnout therefore posing a threat to patient safety (Li et al.).

A high risk of burnout syndrome can also affect new types of workers such as organ donation coordinators, who travel between hospitals in search of potential organ donors that meet the criteria for brain- or heart-related deaths. As a link between organ donors and recipients, these professionals are also responsible for communicating with the families of patients; to help convince them to donate their organs so that potential organ donors can easily become actual donors. Currently, most organ donation coordinators suffer from varying degrees of anxiety, depression, and insufficient sleep due to constant work pressure (Luo et al.).

Cross-cultural research has also indicated a high incidence of burnout syndrome in social workers; another occupational field that faces several challenges. Social workers are generally called upon to perform administrative tasks, client visits, client contacts, case work, case management, teamwork, program planning, program implementation, community networking, evaluation, supervision, and training; however, their job identity has not yet been fully established, and the public often misperceives social workers as volunteers. The absence of adequate professional recognition often deprives social workers of professional respect, which affects their ability to perform their job to the best of their ability (Tuo et al.).

Burnout is not a phenomenon specific to only the healthcare sector, but extends to other jobs. Firefighters, for example, are frequently exposed to traumatic events and stressful situations, increasing their vulnerability to burnout and psychiatric conditions such as anxiety and depression. As first responders, firefighters have to deal with constantly evolving tasks including fire suppression and rescue services, which may cause serious injury or death. Witnessing long-lasting events and tragedies that endanger the lives of colleagues can negatively affect an individual's mental and physical health, causing anxiety and depression. Without sufficient external assistance and organizational support, the mental health problems faced by firefighters become a challenging problem (Tao, Liu et al., Tao, Ma et al.).

Instant Delivery Service (IDS) workers are also considered to be at a high risk of work-related stress and burnout. This group of workers are responsible for delivering essential goods to residents during the pandemic, ignoring the risks to their health, completing much of their work outdoors with considerable exposure to adverse weather conditions, air pollution, and accidents. The demand for quick deliveries and the payment-per-delivery philosophy of some companies create additional stress that increases the risk of unsafe behaviors and accidents. Furthermore, IDS workers are temporarily employed and poorly paid, often receiving wages by the hour or by the number of deliveries. This tends to induce an intense work pace for extended time periods without breaks, as well as increased work stress, fatigue, and burnout (Chen et al.).

Finally, certain groups of workers such as deep-coal miners could experience unreasonable labor organization, failure to enforce rules, and inadequate technical specifications; these could become risk factors for job burnout (Yang et al.).

In conclusion, the high prevalence of burnout in several occupational contexts significantly impacts public health outcomes and health services, highlighting the need to initiate policies such as work-life balance in order to alleviate high physical workloads and to provide support to make emotional workloads more manageable. Significant importance should be placed on the cultivation of psychological resilience and the wellbeing of all workers, particularly those with chronic diseases, in order to improve their adaptability and ability to cope in the face of adversity (13). Specifically, workers should be given time to rest so that they can engage in self-regulation. They should be encouraged to cultivate their interests and hobbies in their spare time. Psychological training courses should be established for workers (Luo et al.). Redistributing the demand for mobility by, for example, expanding and diversifying the start times in workplaces, public offices, schools, and shopping centers, can be considered as an effective additional strategy to improve organizational wellbeing, to reduce burnout in several work settings, and to foster greater environmental sustainability with regard to transportation (14). It appears to be necessary to improve awareness regarding this topic, as numerous gaps in knowledge still exist. Despite over half a century of research on occupational burnout, little is known about its prevalence, etiology, treatment, or prevention. The lack of consensus on the nature of burnout has led to a proliferation of definitions and measures of the construct, precluding a reliable estimate of both its incidence and prevalence and adversely affecting the quality of research on this topic (15). Many studies on burnout have limited their analyses to emotional exhaustion, or have not reported findings on cynicism, personal accomplishment, or global burnout. In addition, most of these studies had a cross-sectional design, whereas well-conducted prospective studies could more appropriate for investigating the possible consequences of this syndrome. This is because these types of studies enable identification of the temporal relationship between the exposure (burnout syndrome) and the outcomes (consequences) (16, 17).

Finally, and particularly during the COVID-19 pandemic, there is an urgent need for policymakers to recommend applicable and appropriate burnout prevention strategies so as to ensure a healthy work environment for the healthcare force. Although HCWs rely heavily on the training and equipment provided by their organizations, managerial support and effective leadership must also contribute to the avoidance and mitigation of adverse psychological outcomes for HCWs (Aljiabri et al.).

## Author contributions

AS and LV contributed in conceptualization and methodology. AS reviewed the literature and wrote the first draft. LV and JA-W reviewed and edited the final version of the manuscript. All authors have read and agreed to the published version of the manuscript.

## Conflict of interest

The authors declare that the research was conducted in the absence of any commercial or financial relationships that could be construed as a potential conflict of interest.

## Publisher's note

All claims expressed in this article are solely those of the authors and do not necessarily represent those of their affiliated organizations, or those of the publisher, the editors and the reviewers. Any product that may be evaluated in this article, or claim that may be made by its manufacturer, is not guaranteed or endorsed by the publisher.
